# Development of iGrow: A Curriculum for Youth/Adult Dyads to Increase Gardening Skills, Culinary Competence, and Family Meal Time for Youths and Their Adult Caregivers

**DOI:** 10.3390/ijerph15071401

**Published:** 2018-07-03

**Authors:** Jade A. White, Rebecca L. Hagedorn, Nicole L. Waterland, Makenzie L. Barr, Oluremi A. Famodu, Amy E. Root, Adrienne A. White, Sarah E. Colby, Lisa Franzen-Castle, Kendra K. Kattelmann, Melissa D. Olfert

**Affiliations:** 1 Human Nutrition and Foods, Division of Animal and Nutritional Sciences, Davis College of Agriculture, Natural Resources & Design, West Virginia University, G027 Agricultural Science Building, Morgantown, WV 26506, USA; jade_white@my.uri.edu (J.A.W.); rlhagedorn@mix.wvu.edu (R.L.H.); mbarr6@mix.wvu.edu (M.L.B.); oluremifamodu@tcomn.com (O.A.F.); 2 Division of Plant and Soil Sciences, Davis College of Agriculture, Natural Resources & Design, West Virginia University, Horticulture, 3315 Agricultural Science Building, Morgantown, WV 26506, USA; Nicole.waterland@mail.wvu.edu; 3 Department of Child Development, College of Education and Human Services, West Virginia University, 709B Allen Hall, Morgantown, WV 26506, USA; amy.kennedy@mail.wvu.edu; 4 School of Food and Agriculture, University of Maine, 5735 Hitchner Hall, Orono, ME 04469, USA; awhite@maine.edu; 5 Department of Nutrition, University of Tennessee, 1215 W. Cumberland Avenue, 229 Jessie Harris Building, Knoxville, TN 37996-1920, USA; scolby1@utk.edu; 6 Nutrition and Health Sciences Department, University of Nebraska-Lincoln, 110 Ruth Leverton Hall, Lincoln, NE 68583-0806, USA; lfranzen2@unl.edu; 7 Department of Health and Nutritional Sciences, South Dakota State University, Box 2275A, SWG 425, Brookings, SD 57007, USA; kendra.Kattelmann@sdstate.edu

**Keywords:** curriculum, gardening, nutrition, education, fruit and vegetable, youth

## Abstract

This manuscript describes the development of a “learn by actively participating” curriculum for youth and their adult caregivers (dyad pair) to increase gardening skills, culinary competence, and family meal time. The curriculum was developed by integrating “iCook 4-H” and Junior Masters Gardener “Health and Nutrition from the Garden”, and “Essential Elements of 4-H Youth Development” curriculums with additional resources for gardening activities from the USDA’s My Plate and garden-based recipes. Expert reviewers (*n* = 11) provided feedback on the curriculum content, session structure, dosage, age appropriateness, and balance of the three focused areas. Seven family dyads (*n* = 14) participated in focus groups about understanding of need, interest, barriers, and potential engagement. A 10-week curriculum was developed and named: iGrow. The curriculum is a hands on, active learning program delivered through five, two-hour sessions using a family dyad model. Three main focus areas included gardening, culinary skills, and family conversation/interaction that all focused on togetherness. For the final iGrow curriculum, expert-level content review and feedback from focus group dyad pairs was used to revise the curriculum which further enhanced the approach and balance of the curriculum content. Focus group feedback supported appropriateness, dosage and learning objectives, and content depth. This curriculum has been developed to provide knowledge of gardening and culinary skills with the goal of increased consumption of fruit and vegetables.

## 1. Introduction

Despite decades of nutrition education, American youth continue to consume fewer servings of fruits and vegetables than the national recommendations of five servings per day [1,2]. It is estimated that only 30% of youth report consuming the recommended two or more daily servings of fruit and only 7% report consuming the daily-recommended servings of vegetables [3,4]. Fruit and vegetable consumption in youth is associated with taste preferences, availability, and parental consumption [3,5,6]. When there is low availability in the household of fruit and/or vegetables, exposure to these foods is decreased which can result in a lower taste preference of fruits and vegetables [7], as well as being associated with a lower intake [8].

Recently, to promote fruit and vegetable intake, researchers have combined intervention methods on nutrition and cooking with gardening programs, by incorporating gardening during and after-school programming [9,10]. Gardening curricula commonly apply what youth are learning in the classroom by using hands-on experience through growing and producing fruits and vegetables. School gardening curricula may be the ideal strategy to increase fruit and vegetable intake in youth as it allows for hands-on opportunities and repeated exposure to the food [11]. There is anecdotal evidence to support the implementation of school gardens and their potential benefits to increase health, but intervention results have been are mixed and use varying measurement tools. These limitations make conclusions difficult to draw as to whether fruit and vegetable intake will be increased by participating in gardening curriculum [10]. Further, many of these studies are flawed with weak study design—including convenience sampling, self-reported fruit and vegetable measures, short study period, and small samples—making the need for a curriculum tested with rigorous study design essential [12]. 

Dietary habits are similar within families, with child fruit and vegetable consumption mirroring that of their parents [13,14,15]. Intervention programs that are designed with parental involvement may be more likely to be successful as parents are the gatekeepers for purchasing and preparing food in the home environment [16,17]. If parents do not value the benefits or the importance of eating fruits and vegetables then youth intake can remain below recommended amounts [14,18]. Therefore, interventions aimed at promoting fruit and vegetable intake have found that parent involvement, along with the child, in health programming results in a greater likelihood of behavior change [19]. 

To our knowledge, there is no dietary intervention curriculum targeted to youth and caregiver dyads to increase fruit and vegetable intake by gardening, cooking, and nutrition education combined approach. This combination of behaviors was chosen to target, due to the multifaceted nature of health behavior and lifestyle, with gardening, culinary activity, and family meal-time all being shown to be associated with fruit and vegetable intake and can impact health throughout the lifespan [20,21,22,23,24]. The iGrow curriculum was developed using establish curricula to incorporate the dyad model for out-of-school programs with family interaction through gardening, cooking, and eating together. Youth and adults utilize experiential learning, which facilitates application of knowledge as part of the iGrow curriculum. Specifically, families develop health promoting skills together by learning how to grow their own produce through container gardening, creating recipes together, and experiencing the importance of family meals.

The goal described here was to develop a ‘learn by actively participating’ curriculum using evidence-based programs to increase gardening skills, culinary competence, and family meal time for youth (early adolescent years) and their adult caregiver (dyad pair). The objectives were to (1) describe the process of curriculum development, (2) conduct formative evaluation on curriculum components, and (3) identify stakeholder feedback about health needs and barriers to a dyad gardening/culinary curriculum and strategies to overcome barriers. 

## 2. Materials and Methods

### 2.1. Study Design

Content for iGrow was developed using three evidence based curricula; a Junior Master Gardener program titled “Health and Nutrition from the Garden” [25], “iCook 4-H” [26,27], and “Essential Elements of 4-H Youth Development” programs [28]. The curriculum was refined through a formative review by experts in different health, education, and horticulture fields to obtain qualitative and quantitative data about the appropriateness of the curriculum and to identify if the objectives of the curriculum were being met within each session. The final phase of development consisted of additional refinement to determine the appropriateness of the curriculum for the target audience and evaluate the objectives of the sessions through qualitative and quantitative methods. West Virginia University Institutional Review Board exempted the expert review survey and approved the focus groups. Expert review occurred in Spring 2015. Focus groups were conducted in Spring and Summer 2015. This study was conducted in accordance with the Declaration of Helsinki and all subjects gave their informed consent for inclusion before they participated in the study. 

### 2.2. Curriculum Development and Theoretical Support

The curriculum structure and objectives were grounded in the social cognitive theory (SCT) [29]. The SCT, in terms of health promotion, is based around the notion that health is influenced by an interaction of environmental, personal, and behavioral variables [30,31]. The SCT incorporates personal, environmental, and behavioral factors in many disciplines of behavior change. Specifically, in regards to this manuscript, SCT can be used to promote healthier lifestyles and to influence sustainable behavior change [32]. These constructs were addressed in the development of the curriculum (Table 1). During the development of the curriculum youth age, socioeconomic characteristics, and gender of participants were taken into account to ensure that the curriculum was appropriate for multiple environments and populations.

The development of iGrow involved incorporation and modification of the three evidence-based curricula; “iCook 4-H”, “Health and Nutrition from the Garden”, and the “Essential Elements of 4-H Youth Development” programs. The “iCook 4-H” program was used as the framework for the session layout structure, the cooking/nutrition information, and the family meal time/goal setting. Family goal setting was also included for families to have the opportunity to set healthy goals and work towards them as a team. iGrow is an extension of the “iCook 4-H” program and expands the program from cooking and family meal time by including gardening and using containers to grow fruits and vegetables. The “Health and Nutrition from the Garden” program was modified to develop the gardening component of the curriculum. Activities were included to teach nutrition and health knowledge from the garden and also provide instruction on how to take care of a garden. Supplemental session plans were also used from Extension sites to teach the gardening concepts and methods. Lastly, the “Essential Elements of 4-H Youth Development” contains four elemental concepts that have seen to be essential in youth programming to achieve the most effective learning environment. The four concepts include belonging, independence, mastery, and generosity that creates an environment that facilitates positive youth development and allow youth to learn experientially, facilitate a safe environment for learning, and allow for mastery of skills and empower youth to affect positive change in their environment [33]. These elements were incorporated into the curriculum throughout all five sessions. 

iGrow is comprised of five sessions that are approximately two-hours long per session. The iGrow program has three main objectives of container-gardening, cooking, and eating together as a family. Dyads follow a step-wise process to learn skills needed to grow produce in containers, empower youth to make their own healthy recipes, and give families the tools to have more regular family meals. iGrow was created for 9 to 10-year-old youths and their primary food preparer with flexibility to be taught by Extension staff, graduate student, or an interested member of the community. It is also ideal to have assistants such as undergraduate or high school student volunteers to help facilitate the class. iGrow can function anywhere that has access to adequate sunlight and cooking space. Sessions include optional activities and information that can be used based on time and space. Each iGrow session consists of six parts with each part equally contributing to the objectives and experiential learning. The six parts include: (1) icebreakers, (2) set activity, (3) gardening activity, (4) cooking activity, (5) family meal time, and (6) goal setting. Table 2 shows a breakdown of each session.

### 2.3. Expert Review

Experts in the fields of nutrition, horticulture, gardening, curriculum development, youth/family development, and 4-H leadership training were utilized to collect feedback about the curriculum. The sample was based upon expertise in different fields and was therefore a non-randomized sample. Experts (*n* = 29) were contacted through email and invited to participate in the review process of the curriculum by completing an online survey. A $25 gift card was offered as an incentive for completing the review. Those who agreed to participated (*n* = 17) were instructed to read through the curriculum and answer the survey questions that were coordinated with respective sections. 

Experts completed a 35-item survey online via Qualtrics (see Appendix A), read over each session of the curriculum, and reported on how clear the objectives were, provided comments on the gardening and cooking component, and gave feedback on areas of likes, dislikes, and any confusion within each session. Questions were open-ended, interval, multiple-choice, and multiple-response options to collect formative feedback on the curriculum. Interval questions were on a scale of 0–10 with higher scores representing more positive response to the curriculum. Data were collected on whether the curriculum covered the objectives that were set forth in each session, if the sessions were occurring in sequential order, and if the resources provided were appropriate for running the program. The reviewers were also asked how well the curriculum would work in their community, and if they thought gardening, cooking, and family meal time would be increased after families participated in the program. The open-ended format was for responses about what was easy and difficult to understand about the curriculum, what their opinion was about the resources provided, and any additional feedback they thought would be helpful.

### 2.4. Focus Groups

Stakeholders (adult caregivers—including mother, father, or grandparent—and youths aged 9–10) in the community were contacted to participate in focus groups. Focus groups occurred to enhance the understanding of the community’s perceptions around gardening, cooking, and eating together as a family. Feedback was then integrated into the curriculum to benefit the community environment and health needs that were identified by stakeholders [34,35]. Recruitment of stakeholders occurred by identifying families who had previous involvement in family programming and asking them to aid in the recruitment process. The community members who were identified (*n* = 6) were asked to distribute a flyer to other families in the community who they thought would be interested in participating. A flyer was also distributed to a local elementary school in third and fourth grade classrooms (*n* = 30) inviting families to participate. Focus group (*n* = 14) participants were given an incentive in the form of a tomato plant and a $25 gift card for their time. 

To facilitate a comfortable environment, before the focus group began the participants, facilitator, and note takers all completed an ‘icebreaker’ activity that encouraged the participants to become more familiar with one another. After the icebreaker, adult caregivers and youth were separated into different rooms. Each focus group was comprised of one facilitator and one to two trained note taker(s). The three main themes of the focus group included: gardening knowledge/comfort, cooking knowledge/comfort, and family meal enjoyment/occurrence. The outline of the questions is listed in Table 3.

### 2.5. Analysis

From the formative evaluation, expert review responses were analyzed using descriptive statistics and thematic analysis based on the question type [36,37]. The averages and frequencies were determined for the quantitative data. The qualitative data from the open-ended questions were analyzed using thematic analysis, following Braun and Clarke’s methodology by two independent reviewers [38]. In support of the thematic analysis methodology, feedback was coded based on identified themes. Common themes were identified to determine and include suggestions from the expert reviewers. Thematic analysis was also conducted to analyze the qualitative feedback from the focus groups. The data from the youth and adult focus groups was transcribed into a word document then compared between the different note takes. The data were then coded, reviewed, and compared to each focus group. Each question was analyzed to determine themes and sub-themes that emerged from the discussions.

## 3. Results

### 3.1. Expert Review Sample

The expert review was completed by 11 of the 17 reviewers that agreed to participate (64.7% response). The experts included professionals in one or more of the following fields: nutrition (*n* = 7), horticulture (*n* = 1), curriculum development (*n* = 1), youth/family development (*n* = 1), and Extension/4-H youth programming (*n* = 2). The average amount of years the reviewers reported working in their field was about 13 years (2–30+ years). The average time spent reviewing the curriculum was about 40 min, with the minimum time being 15 min and the maximum time being 125 min.

### 3.2. Expert Review Results

The results for the quantitative questions were analyzed in Excel using the mean and median formulas. The average score, when asked how well the curriculum was understood, was 8.82 out of 10. Ninety percent of the reviewers replied that the curriculum overview was clear regarding the materials needed to run the program. Majority of the reviewers (80%) reported that all five of the sessions in the curriculum were appropriate in covering the session objectives, with the exception of session three which had a lower percentage (75%), agreeing that the session objectives were appropriately covered. When asked how feasible it would be to run the program in the reviewer’s community, the mean score was 7.82 out of 10. When looking at whether reviewers thought the curriculum would increase skills, culinary skills had the highest mean score 7.45 followed by a mean score of 7.36 at increasing gardening skills, and lastly a score of 6.73 at increasing family meal time. Results are shown in Table 4.

#### 3.2.1. Session 1

What’s Growin On? Common responses for this session focused on the gardening component. Responses included the need to give more details on how to garden and the possible importance of having the families take home their own plant and grow at home along with the program. This was suggested to keep the families engaged in the learning objectives of the curriculum in-between sessions. Another common theme was streamlining the curriculum to ensure that all sessions were in the same format.

#### 3.2.2. Session 2

Washin’ and Wormin’: Reviewers suggested making the objectives more clear and measurable throughout the session and stay away from objectives that start with ‘learn’. Composting worms was introduced in this session and it was suggested that participants take home their own worms to continue the learning process at home and report back on their progress at the next session. Time was a theme that emerged with this session, and it was suggested that the time allotted may not be enough. 

#### 3.2.3. Session 3 

From Farm to Table to Yummy: Feedback for this session centered around keeping the sessions connected to each other and focused more on outside of the session and at home activities that should be incorporated. Reviewers thought that it was important to have the family meal time be more interactive and to facilitate discussion on how the dyad pairs can spend time together at home engaging in healthy activities. Time continued to be a theme in the feedback and it was mentioned that there may not be enough time between sessions to see enough growth of the plants to accomplish the gardening component in Session 3, which is transplanting. 

#### 3.2.4. Session 4 

Fiber Fuel: Common themes that emerged in the feedback for this session included rewording the objectives to make them clearer, talk more about the safety component of gardening, and include more details for the activities in general. Referencing to the multiple icebreaker options, reviewer 6 commented that, “while it is important to observe the group and provide the correct activity, you may want to recommend a specific activity to go along with each lesson”. 

#### 3.2.5. Session 5 

Growing, Growing, Gone! The feedback on this session focused again on timing, but also on streamlining the curriculum so that the directions in each section are the same throughout. Comments from the reviewers were positive in that they thought the activities covered in this session would be good learning experiences for both the youth and adults participating in the program. One reviewer suggested that an activity should be altered to focus more on fruits and vegetables rather than an activity that used a meat-based protein to taste test different spices, and to have the youth and caregivers “taste test different (uncommon) fruits and vegetables and to determine if they were ripe/unripe”. 

#### 3.2.6. Overall Opinion of the Curriculum 

Comments that occurred frequently included that the curriculum was nicely laid out, clear, and that each session was comprised of multiple parts of gardening, cooking, and family meal time. Another common theme about the overall curriculum was about the materials provided. Reviewers requested more materials in the beginning of the curriculum to limit confusion about how to prepare to run the class and what materials/supplies needed to be set up. Another common theme was focused around time-oriented questions. Questions arose about how much time was needed between the sessions, how much time each session would take, and how many months ahead of time are needed to prepare to run the program. Overall, the expert reviewers requested more details about cost, time commitments, and overall purpose of the curriculum.

### 3.3. Focus Group Sample

Two adult and two youth focus groups were conducted. All of the adults (*n* = 6) in the focus groups were women, married, had at least one child, and were living in the same community. The youth (*n* = 8) who participated were all in elementary school and were a mix of males (*n* = 3) and females (*n* = 5). The majority of the children had little to no experience with either gardening and/or cooking and the caregivers were equally diverse in skill level with gardening and cooking. 

### 3.4. Focus Group Results

#### 3.4.1. Gardening 

Youth were able to name the fundamental components necessary to make a garden. Common supplies listed by youth were land, water, tools, and seeds. Youth were not as sure about how much land you would need, one participant commented that “acres and acres of land are needed to have a garden”, but another commented that “(you) don’t need too much space for a cucumber garden”. Questions that arose from the discussion were based around how much sun, water, soil, and land were needed to establish a garden and youth were unsure on the specifics of garden care. 

When discussing how difficult it would be to take care of their garden all youth agreed that it would be a difficult task. Common themes included that it was difficult to know how much sun the plants should be getting or how much to water the plants. The majority of the youth said that they “would need a lot of help with taking care of the garden” and also commented that it would be “easier if my mom helped me” take care of the plants. When asked to picture their perfect garden many of the participants commented that they would want to grow “cherries, berries, carrots, tomatoes, broccoli, and spinach”. Many youths stated that they would grow food that they would want to eat and said “I would eat all the food I grew”. Some youth also said that they would grow the food for their family or to sell to their community. Another common theme in the garden was that it would have to look nice. One participant said, “I would make it look pretty” and “I would decorate my garden with flowers because it would look cool”. Making sure that the garden was producing food that they liked to eat and that it looked aesthetically pleasing were the two main priorities and motivators when thinking about their ideal garden environment.

Caregivers reported many similar themes when discussing the benefits of having a garden at home. The most common theme that arose was taste differences that the participants noticed when eating home grown vegetables. One participant stated that “the taste is always much better and that makes me want to eat more” and that the “quality is better which makes the taste better”. Another main theme was decreasing preservatives and increasing health. Common responses from participants included that “health is the number one benefit to the home garden. Controlled environment is key—no preservatives” and “you don’t have to worry about the ingredients. You don’t need to wash it because it’s organic”. Caregivers also noted the convenience of having the ingredients in the home and having easier access to fresh produce.

When talking about what would make the participants uneasy when trying to take care of their home garden, the most common theme was the logistics of the gardening process. Participants felt uncertain about when the right time would be to start their seeds, how they would identify them later, and how to prepare themselves for the whole process of taking care of their plants. Another logistic issue brought up was having enough space to garden. One participant commented that, “space would be an issue—I live in an apartment. There is no room for an ideal garden”.

When talking about gardening with their child, the majority of the caregivers discussed both pros and cons about gardening together as a family activity. The main theme that emerged was that it depended on the child’s personality. One caregiver commented that her son did not like to get his hands dirty, and another comment was that, “it would depend if my child was listening to me or not”. Another caregiver shared that they thought it would be fun and have heard of other families who have gardened together, and it looked like a good project, they also stated that, “my child is an extrovert that enjoys making things look lovely and seeing her work done”. Lastly, it was mentioned that gardening as a family “could be a fun and nurturing experience”. Overall, the caregivers reported that it depended on the child if gardening would be a good family activity to be involved in. 

#### 3.4.2. Cooking 

When discussing tools needed in the kitchen, youth listed common tools such as knives, spatulas, cutting board, measuring cups, and utensils. Another main necessity that the majority of youth said was that a recipe was needed to cook. 

The two themes that emerged when talking about what was easy about cooking, included that it was easier to cook when you were following recipes and when you had more experience cooking. One participant stated, “I don’t practice cooking, I’m not a star at it” while another participant said, “that cooking is easy, but my friends that don’t cook would think cooking is difficult”. When talking about following a recipe, one participant commented that, “it’s easier to follow a recipe step by step”. Youth also commented that recipes were easier to follow when fewer ingredients were needed and it was easier for themselves to cook “easy things” that had fewer ingredients. 

When discussing difficult aspects of cooking the most common theme was measuring out the ingredients and following the directions correctly. The majority agreed that reading a recipe could be difficult, as one stated, “making sure you don’t heat things up too much” could be challenging and as another said, “if you put something in the (oven) too long, then it’s not good”. When it came to measuring, all the participants said it was difficult to measure correctly, and one youth commented that, “at first I did not read the measurement right, I would think that ¼ cup is the same as 1 cup”. The participants also talked about how certain safety guidelines need to be followed in the kitchen. Many youths reported that knife safety was important and people, as one youth put it, “need to be careful not to cut their fingers with knives”. Cross-contamination was also alluded to in multiple comments when youth said that “you can’t cut carrots and steak on the same cutting board” and “can’t cut all the fruits and vegetables on the same board”. While all the information or instructions that youth discussed during the focus group may not have been correct, the majority of the time they were on the right track to give appropriate guidelines for someone who was trying to cook. 

The next question focused on healthy eating and aimed to gain insight on what youth considered healthy and non-healthy foods. Common foods that were mentioned as healthy from youth included fruits, vegetables, rice, chicken, milk, and cheese. Multiple participants commented on eating organic foods and that “organic foods are healthy” and another commented that, “organic means health. Another common theme was moderation. Many participants commented that eating too much of anything could make people unhealthy, and that ‘good stuff’ could become bad for you”. A few of the participants mentioned that it depended on how certain foods were cooked and that “deep-frying is bad” and that “vegetables are deep-fried then they are not good for you”. Some participants thought that staying away from bread would help someone be healthier and contrary to that one participant stated that, “bread, cereal, rice, and garlic bread are healthy (for you)”. Three of the participants referred to My Plate and that a balanced diet of honey, carrots, protein, grain, fruit, vegetables, and dairy were all healthy. Overall, the majority of youth agreed that eating a balanced diet was important to stay healthy. 

When caregivers were discussing how they decided what they would have for dinner, time was the biggest theme that emerged, with a sub-theme of health. Time played an important role in coming up with dinner ideas based on ingredients that were on hand or if they had to depend on a fast-food option. One participant stated, “that picking up a second job caused more of a dependence on fast food”. The majority of participants mentioned they had started to plan out meals and got organized with what they were eating during the week to make the process easier. Other caregivers noted that taste and food preferences played a role when deciding what meals would consist of and it could be challenging when there were picky eaters within the household. 

Caregivers discussed how their children helped them cook and challenges they face when trying to involve them in the cooking process. One participant shared that her son was an excellent cook and her daughter had noticed how the family had been cooking and wanted to learn more about the process. Another caregiver also commented that, “as a mother, you need to learn how to let go because giving them (child) a knife can be a scary thing”. The majority of participants agreed that they would be open to their children helping in the kitchen but would need to be present to feel comfortable with their child cooking. When discussing barriers participants faced when cooking with their child, skill level was a common theme. The caregivers agreed that their children liked to experiment in the kitchen. One participant shared that “I like things done right, my way, and in order”. Time was also a common issue. One participant commented, “no, he does not help me. I always cook when he is doing homework. I teach him when time is available”. 

Caregivers reported that the best way to talk about fruits and vegetables was when their child was eating, but majority reported that it was difficult to try new foods. One caregiver suggested teaching about how taste buds changed and said her child “now (they) will come and say ‘can I have a piece of that?’ He learned from a program that you need to try something 12 times before you decide not to eat it”. Another caregiver shared that her child was very conscious of weight and that resources had helped a lot with determining healthfulness. It was clear from the feedback that limited dialog was taking place in the home about fruits and vegetables and picky eaters posed an issue when trying to introduce new food.

#### 3.4.3. Family Meals 

During the family meal discussion, the youth reported various feedback on when they ate meals together. About half of the youth reported that they always had family meals at dinner and then the other half reported that family meals only occurred on special occasions or on the weekends. The location of the meal was also mixed. One of the youth participants stated that “sometimes we sit in the TV room and sometimes in the dining room”. Other participants verbally agreed that they sometimes sit and have meals with their families on their couch, while others stated that they had to be sitting at the table. One youth reported that their family ate dinner together “mostly everyday but it depends how my brother and sisters feel. If they are mad, they don’t eat with us.” Dinner was the most popular meal to have as a family, and then breakfast on the weekends only. Family interaction was also seen to be an important factor when it comes to having family meals. 

Participants discussed different aspects that made family meals enjoyable and not enjoyable. The most common occurrence was that conversation played a major role in whether the meal was a good experience. Youth said they “don’t want arguments”, “parents are distracted, and I eat in silence”, and “too much work talk” can all make family meals not enjoyable. Conversation that was said to be enjoyable was when youth talked about their day at school or could listen to other conversations that were occurring at the table. Participants also stated that food played a major role in the meal and that if the food was good then the meal was more enjoyable. A participant shared that, “my sister always makes salads. She puts a lot of weird things (in it). It’s good and bad.” Another youth stated that “two bites per plate is the house rule” and similar statement was that “I usually taste everything”. 

It was also common, among the participants, that there was background noise during the family meal. The majority of the participants stated that either the TV was on or the computer was on during the meal. Youth also agreed that they enjoyed watching TV while eating. A few of the participants shared that they were not allowed to watch TV or use the computer during the meal, but this was not the norm within the focus groups. From the discussion it was understood that both family interaction and food were key factors in family meals, and that background noise was common during meal time.

All of the caregivers reported that they made an effort to try and have regular family meals, but the most common barrier was time constraints. It was shared that when the children were younger family meals were easier, but now with different schedules family meals were more difficult. It was also a common theme that family meals were easier during the weekend when they were not rushed. Participants shared that they tried and had meaningful discussions at meal time and “try to find something fun to talk about”. One caregiver shared that their family meal was not always at the dinner table and sometimes they would eat on sofas “depending on what the meal is”. The majority of the participants said that they liked to use family meals to talk about the day and catch up with one another. 

When discussing what makes family meals enjoyable, the participants all commented that positive conversation and positive attitudes were important components. Common remarks included “the best meals we have are when everyone is involved”, “we try not to bring negative conversation during dinner”, and “we talk about the day and find out what everyone is doing”. Another comment was that it was helpful to use paper products to minimize the cleanup. One aspect that was mentioned, that made meals less enjoyable, was having picky eaters. It was brought up that when children were very vocal about what they disliked it could make for a less enjoyable meal. 

Half of the caregivers reported that it was common for there to be background noise when eating a family meal. One participant shared that “the TV is in the living room. It has become a problem. We usually have meals in the living room not the kitchen”. Some caregivers mentioned other distractions such as animals, emails, and schedules that were distractions during family meals. From the discussion the feedback showed that habits, such as watching TV during dinner, were difficult ones to change.

## 4. Discussion

The aim of the study was to develop a curriculum focused around gardening skills, culinary competence, and family meal time called iGrow. From three existing evidence-based curricula, iGrow was designed for early adolescents and their caregivers and underwent expert review and stakeholder focus groups to gain formative evaluation feedback on the curriculum prior to pilot testing in the future. The curriculum was generally supported by reviewers and community participants to provide knowledge of gardening and culinary skills.

Currently, there are a few garden-based nutrition education programs for youth, predominately in the school setting. “Nutrition to Grow On” [39] and “Nutrition in the Garden” [40] are two examples of evidence-based gardening curriculums that are currently available. “Nutrition to Grown On” has been evaluated for improvements in elementary student’s nutrition knowledge and vegetable preferences, while “Nutrition in the Garden” participants showed increased fruit and vegetable consumption [41]. Like both of these curriculums, iGrow focuses on the impact gardening can play in living a healthy lifestyle through nutrition while also weaving in physical activity and goal setting. However, both curricula are school-based and lack translation into the home environment. Further, incorporation of the adult caregiver is missing, diminishing the chances of sustainable change at home [16]. The iGrow curriculum facilitates this by giving the youth control in the whole planting process, from sowing the seed to harvesting the produce, with the adult assisting the child in the gardening process and food intake at home. This is essential as growing fresh produce has been seen to empower youth, puts them in charge of making healthy choices for themselves, and helps them value healthy eating [42], with further benefits from parental engagement. The notion that parental involvement is important for the success of a youth gardening curriculum was highlighted by youth participants in this current study. Gardening was considered a difficult task based on youth comments and they found benefit in having parental guidance. iGrow curriculum is developed to overcome the shortcomings of past curriculums, to provide a robust curriculum combining gardening, culinary competence, and family meal time for dyads. This curriculum is able to address the barriers youth and adult face to living a healthy lifestyle, as evidenced by the focus group results, in terms of gardening, cooking, and family meals. 

After reviewing the responses from experts as well as youth and adult focus groups, participants supported the use of the iGrow curriculum in the community, but the discussions also brought to light issues that needed to be revised within the curriculum. First, time to deliver each session was expanded from an hour and a half to two hours. Increasing the delivery time of the session should allow the leader to have more time to cover all the details of the session and also have adequate transition time between sections of individual sessions. Time was also a question when it came to having plants ready to be transplanted, harvested, and how much time there would be between sessions. This issue was also addressed in the revised curriculum explaining that the leader of the curriculum would need to have plants already growing to be able to accomplish the session activities. It is explained in the revised curriculum that plants should be started six weeks ahead of time to be ready to transplant by the third session. Time in between sessions was still left to the discretion of the leader of the program. Ideally, sessions would be held a week apart from each other, but in actuality this may not be possible. Secondly, revisions were made to the curriculum that focused more on encouraging the activities that were happening during the session to also occur at home. The revised curriculum has the families planting seeds that they will care for at home and challenges them to bring in pictures and report on how their plants are doing. Lastly, from the focus group feedback, it was found that television during dinner was an important topic that was omitted from the original curriculum. After conducting the focus group, helping families become comfortable with the topic of turning the television off during dinner is now covered in the family meal time component of the curriculum. 

Limitations were experienced during the curriculum development process. The sample of both the experts and focus group participants were based on a convenience sample. The expert reviews of the development were not equal in all disciplines with limited number of reviewers in certain expert fields. Ideally the sample size of experts in each field would be larger to determine more saturated expertise feedback in the different focused areas of the curriculum. This could have attributed to the variability that occurred in reviewer scoring, with some expert areas not being as reviewed as others. Another limitation was number of participants in the youth and parent focus group. A small percentage (20%) of individuals invited to participate followed through and came to the focus group with each focus group replicated twice because of limited subjects. This limited sample could have prevented data saturation from occurring. Additionally, replication of the study with more rigorous qualitative methods could improve study results and further pilot testing of the curriculum is needed to evaluate the effectiveness in meeting curriculum objectives. This curriculum should be tested with a rigorous design—including randomization, control group, and minimum one-year study period—to improve the literature on garden curriculum and fruit and vegetable intake. 

## 5. Conclusions

Increasing fruit and vegetable intake in youth is a complex, multi-facetted public health issue facing society. iGrow is a five-session youth and adult out of school program that adds to the body of literature on curriculum for improving dietary behaviors in youth through gardening. iGrow was developed from three evidence-based curricula to include robust aspects of gardening, culinary skills, and family meals and further underwent expert and community stakeholder review. The curriculum has been revised from results and needs to be tested in diverse populations to determine if the curriculum can provide positive outcomes for gardening skills, culinary competence, and family meal time as it was designed. 

## Figures and Tables

**Table 1:** Curriculum development grounded within social cognitive theory.

**iGrow Curriculum Foci**	**Personal**	**Behavior**	**Environment**
	Nutrition knowledge	Gardening	Social norms
	Food preferences	Cooking	Food availability
	Perceived barriers/benefits	Family Meals	Positive influence of health behaviors
	Attitudes	Self-efficacy	Social support

**Table 2:** iGrow session overview.

Session	Set Activity	Gardening	Cooking/Nutrition	Family Meal Time	Goal Setting
**WHAT’S GROWIN ON?**	ABCs of plants	Planting seeds	Introduction to basic knife safety, garden salsa	Focusing on family meals	Setting SMART-R Goals
**WASHIN’ AND WORMIN’**	Hand washing	Worm composting	Food safety, salad Recipe	Taste testing	Short Term and Long Term Goals
**FROM FARM TO TABLE TO YUMMY**	How food leads back to plants	Transplanting	Using leftovers and fresh vegetables	Place setting	Setting SMART-R Goals
**FIBER FUEL**	Fiber sources	Watering and fertilizing	Healthy snacking	Family communication	Setting SMART-R Goals
**GROWING, GROWING, GONE!**	Discover spices	Harvesting plants	Using non-meat sources of protein	Positive family communication	Setting SMART-R Goals

**Table 3:** Focus group question outline.

Focus Group Questions		
Focus Area	Caregiver	Youth
**Gardening**	What benefits do you see in having a home garden? If you start to think about building your own garden, what makes you uneasy about the process? Do you think gardening with your child would be a good activity to do together?	If I asked you to explain to a friend what you needed in order to build a garden, what would you tell them? How do you think you would go about caring for your garden? How hard do you think it is to garden? If you could grow anything in your garden, what kinds of plants would you grow?
**Cooking**	When you are thinking about cooking dinner, how do you decide what to cook? Does your child help you at all when cooking? What are you comfortable/not comfortable with your child cooking? If you could get any help with cooking/preparing meals what would it be?	What are tools you need in the kitchen in order to cook? What steps do you take when preparing to make a recipe? What are safety tips you should take when cooking? What makes one recipe healthy and another recipe not healthy?
**Family Meals**	Would you say your family are healthy eaters? What are the barriers when it comes to having a family meal? What do you think are the benefits in having family meals? How could having family meals be easier?	Do you think your family eats healthy? Does your family ever sit down and eat a meal together? What do you like or dislike about family meals? How do you think you could help family meals happen more often?

**Table 4:** Expert review of increasing skills of the overall program.

Area of Focus	Mean	Min	Max	Std	Responses (N)
**Gardening Skills**	7.36	2.00	10.00	2.91	11
**Cooking Skills**	7.45	4.00	10.00	1.75	11
**Family Meal Time**	6.73	4.00	8.00	1.10	11
**Feasibility of Implementation**	7.82	2.00	10.00	2.18	11

## Appendix A. iGrow Expert Review Survey

Thank you for your participation in the process of reviewing the iGrow curriculum. Please answer the following questions with your expertise and area of work in mind. At the end of the survey we will ask for a mailing address in order to send you a gift card to thank you for your participation. Also, there will be a box to mark if you would like to be contacted in the future about updates on the program.

1. Which category or categories best describes your area of work?
  - Horticulture
  - Master Gardener
  - Curriculum Development
  - Family and Youth Relationships
  - Human Nutrition
  - Youth Development
  - Teacher
  - Student
  - Other
2. Area of Expertise:
3. How many years have you been working in your field?

**The following questions will ask you about your overall understanding and thoughts of iGrow. For this section you will have had to read through the iGrow curriculum.**

4. After reading the iGrow curriculum leader guide how confident do you feel that you could teach the program? Rank 0–10 for understanding the curriculum.
5. What was difficult to understand about the curriculum overall?
6. What was easy to understand about the curriculum overall?

**For the next 3 questions review pages 1–6 (Introduction)**

7. Does the overview of the curriculum explain the scope and materials needed to run iGrow? Please Explain.
  - Yes
  - No
8. Were there any areas that were confusing when reading through the introduction of the program?
9. What additional information should be included in the introduction?

**For the next 3 questions please review to pages 7–19 (lesson 1)**

10. Are the learning objectives for the lesson clear and adequately addressed throughout the lesson?
  - Yes
  - No
11. Are the gardening and cooking skills appropriate and occurring in sequential order?
  - Yes
  - No
12. Please provide feedback on the lesson: Likes, dislikes, areas of confusion, etc.

**For the next 3 questions please refer to pages 20–32 (lesson 2)**

13. Are the learning objectives for the lesson clear and adequately addressed throughout the lesson?
  - Yes
  - No
14. Are the gardening and cooking skills appropriate and occurring in sequential order?
  - Yes
  - No
15. Please provide feedback on the lesson: Likes, dislikes, areas of confusion, etc.

**For the next session please refer to pages 33–44 (lesson 3)**

16. Are the learning objectives for the lesson clear and adequately addressed throughout the lesson?
  - Yes
  - No
17. Are the gardening and cooking skills appropriate and occurring in sequential order?
  - Yes
  - No
18. Please provide feedback on the lesson: Likes, dislikes, areas of confusion, etc.

**For the next section please refer to pages 45–55 (lesson 4)**

19. Are the learning objectives for the lesson clear and adequately addressed throughout the lesson?
  - Yes
  - No
20. Are the gardening and cooking skills appropriate and occurring in sequential order?
  - Yes
  - No
21. Please provide feedback on the lesson: Likes, dislikes, areas of confusion, etc.

**For the next section please refer to pages 56–67 (lesson 5)**

22. Are the learning objectives for the lesson clear and adequately addressed throughout the lesson?
  - Yes
  - No
23. Are the gardening and cooking skills appropriate and occurring in sequential order?
  - Yes
  - No
24. Please provide feedback on the lesson: Likes, dislikes, areas of confusion, etc.

**For the next section please refer to pages 68–83 (resources and handouts)**

25. Are the resources helpful with achieving the learning objectives?
  - Yes
  - No
26. What additional resources would be helpful for leaders in order to deliver the curriculum?

**This next section will ask for feedback based on your expert opinion and personal experience.**

27. What do you think would be a good name for this program?
28. On a scale of 0 to 10 what is the feasibility of running iGrow in your community?
29. On a scale of 0 to 10 would iGrow increase gardening skills in youth and parent?
30. On a scale of 0 to 10 would iGrow increase cooking skills in youth and parent?
31. On a scale of 0 to 10 would iGrow increase family meal time for families?
32. Please provide any additional feedback or comments you have about the iGrow curriculum and program.
33. Would you like to be kept updated on the development of the program curriculum?
  - Yes
  - No
34. Would you be willing to participate in a future focus group about the program? If yes, please provide a phone number.
  - Yes
  - No
35. In order to receive your gift card please provide your name and a convenient mailing address so that we can send it to you.
